# Dynamic simulation of management events for assessing impacts of climate change on pre-alpine grassland productivity

**DOI:** 10.1016/j.eja.2021.126306

**Published:** 2021-08

**Authors:** Krischan Petersen, David Kraus, Pierluigi Calanca, Mikhail A. Semenov, Klaus Butterbach-Bahl, Ralf Kiese

**Affiliations:** a Institute for Meteorology and Climate Research, Karlsruhe Institute of Technology, Kreuzeckbahnstraße 19, 82467, Garmisch-Partenkirchen, Germany; b Agroscope Institute for Sustainability Sciences ISS, Reckenholzstrasse 191, P.O. Box 8046, Zürich, Switzerland; c Rothamsted Research, Harpenden, Hertfordshire, AL5 2JQ, UK

**Keywords:** Mountainous grasslands, Biomass yields, Process-based modelling, Growing season, Adaptive management

## Abstract

The productivity of permanent temperate cut grasslands is mainly driven by weather, soil characteristics, botanical composition and management. To adapt management to climate change, adjusting the cutting dates to reflect earlier onset of growth and expansion of the vegetation period is particularly important. Simulations of cut grassland productivity under climate change scenarios demands management settings to be dynamically derived from actual plant development rather than using static values derived from current management operations. This is even more important in the alpine region, where the predicted temperature increase is twice as high as compared to the global or Northern Hemispheric average.

For this purpose, we developed a dynamic management module that provides timing of cutting and manuring events when running the biogeochemical model LandscapeDNDC. We derived the dynamic management rules from long-term harvest measurements and monitoring data collected at pre-alpine grassland sites located in S-Germany and belonging to the TERENO monitoring network. We applied the management module for simulations of two grassland sites covering the period 2011–2100 and driven by scenarios that reflect the two representative concentration pathways (RCP) 4.5 and 8.5 and evaluated yield developments of different management regimes.

The management module was able to represent timing of current management operations in high agreement with several years of field observations (r² > 0.88). Even more, the shift of the first cutting dates scaled to a +1 °C temperature increase simulated with the climate change scenarios (−9.1 to −17.1 days) compared well to the shift recorded by the German Weather Service (DWD) in the study area from 1991−2016 (−9.4 to −14.0 days). In total, the shift in cutting dates and expansion of the growing season resulted in 1−2 additional cuts per year until 2100. Thereby, climate change increased yields of up to 6 % and 15 % in the RCP 4.5 and 8.5 scenarios with highest increases mainly found for dynamically adapted grassland management going along with increasing fertilization rates. In contrast, no or only minor yield increases were associated with simulations restricted to fertilization rates of 170 kg N ha^−1^ yr^−1^ as required by national legislations. Our study also shows that yields significantly decreased in drought years, when soil moisture is limiting plant growth but due to comparable high precipitation and water holding capacity of soils, this was observed mainly in the RCP 8.5 scenario in the last decades of the century.

## Highlights

• Earlier onset of pre-alpine cut grassland growing season entails shifts to earlier cutting dates and more cuts per year.
• Dynamic simulation of cutting dates can tackle these shifts if temperature and soil moisture are considered.
• Climate change increase pre-alpine grassland yields only if growth is not limited by soil moisture and nitrogen.

## 1 Introduction

Permanent grassland cover almost one third of the agricultural land area in Germany and is the dominant land use in the alpine and pre-alpine region of S-Germany (Dierschke and Briemle, 2002; Kiese et al., 2018). In addition to the economic relevance of fodder production for dairy and cattle farming (Sándor et al., 2017), grasslands fulfill a number of other key ecosystem services like water retention, biodiversity, erosion control and soil fertility (Bengtsson et al., 2019; Gibson, 2009). Beside diverse effects of climate change on these ecosystem functions (Hopkins and Del Prado, 2007; Lee et al., 2013; Soussana et al., 2013; Wang et al., 2016; Wiesmeier et al., 2013), productivity is expected to increase in temperate and cold grasslands (Wang et al., 2019) as far as water availability is not limiting (Dellar et al., 2018; Soussana et al., 2013). This effect needs to be examined particularly in the pre-alpine and alpine regions where average warming is predicted to be at a pace twice as high as compared to the global or Northern Hemispheric average (Auer et al., 2007) and will likely accelerate in coming decades (Gobiet et al., 2014; Smiatek et al., 2016). The stimulating effect on plant biomass production caused by increasing temperatures and higher atmospheric CO_2_ concentrations influences future cutting and fertilization regimes (Chang et al., 2017; Soussana and Lüscher, 2007). According to local agricultural practice, farmers cut the grass regularly based on yield demands and maturity stage as influenced by weather and soil conditions (Deroche et al., 2020), thus significant changes in biomass development will likely change the timing of cutting and associated fertilization events throughout the year (Thivierge et al., 2016). Recent climate change has been found to affect species’ phenology in mid- and higher latitudes, especially regarding the earlier onset of spring events with mean global average changes of 2.3 days per decade (Parmesan and Yohe, 2003). Menzel et al. (2020) recently reported an increase of the growing season of +0.261 ± 0.008 days per year and a shortening of the farming season of cropland by −0.149 ± 0.022 days per year in the period 1951–2018 using European plant phenology data.

Modelling studies can help to assess the influence of different management practices, agricultural adaptions (Gómara et al., 2020; Sándor et al., 2018) and future climate changes on the above mentioned key grassland functions by executing long-term climate change scenario simulations (Chang et al., 2017; De Bruijn et al., 2012; Graux et al., 2013; Höglind et al., 2013; Kipling et al., 2016; Owen et al., 2015). The response of grassland productivity and functional diversity to climate change is complex as it implies interactions of weather with soil water and nutrient availability as well as execution of management routines. Fixed annual schedules of management actions derived from current climatic conditions are inappropriate for simulating future grassland productivity under changing climate conditions and are likely to cause bias in simulated grassland productivity. However, due to dynamic changes between years the setup and timing of management events is highly complex and thus was identified as one of the main challenges for model based climate impact studies of grassland ecosystems (Kipling et al., 2016).

So far, many modelling studies on cut grasslands simulated climate change scenarios without an adaption of management (e.g. Abalos et al., 2016; Cordeiro et al., 2019; De Bruijn et al., 2012; Graux et al., 2013; Lazzarotto et al., 2010; Yang et al., 2018). An automatic management routine was first widely used for regional simulations by Vuichard et al. (2007), who integrated dynamic decision rules into the PaSIM model (Riedo et al., 1998, 2000). This algorithm determines cutting dates by maximizing the seasonal dry matter production. It triggers a cut after a minimum of 30 days of regrowth and declining plant growth rates during 10 consecutive days. This approach was adopted for regional simulations by Chang et al. (2015), single site simulations by Gómara et al. (2020) and even for regional climate change assessments (Chang et al., 2017). Another relatively simplistic mechanism for regional simulations was developed by Rolinski et al. (2018) with the dynamic global vegetation model Lund-Potsdam-Jena managed Land (LPJmL). A fraction of biomass is harvested at the end of each month if the aboveground biomass increment was positive since the last harvest. The main focus of these two approaches were Europe-wide regional simulations for which information on real management at this scale was not available. The proposed algorithms were not intended to explicitly simulate and validate shifts in cutting events induced by phenological shifts at the local scale. For a more realistic simulation of the timing of grassland cutting events with climate change, most of the modelling studies conducted so far rule sets based on cumulative growing degree days (GDD) by applying thresholds for accumulated GDD for the first and following cuts (Höglind et al., 2013; Jing et al., 2014, 2013; Thivierge et al., 2016). Results from these studies underline the importance of accounting for additional cutting events (Höglind et al., 2013; Jing et al., 2014) with up to 10 % increase in annual yields using adapted instead of static management for grassland sites in Canada (Thivierge et al., 2016). However, not taking into account limitation of plant growth under drought conditions or stimulation of plant growth by increasing atmospheric CO_2_ concentration can be a disadvantage of only temperature informed GDD based grassland modelling approaches.

Therefore, we present in this study a new dynamic management approach that we implemented in the biogeochemical model LandscapeDNDC (Haas et al., 2013; Kraus et al., 2015), which dynamically provides timing of grassland management under varying climatic conditions. We developed management rules based on long-term comprehensive field measurements of grassland biomass and records of local farmers’ management decisions regarding cutting and manuring events from grassland sites belonging to the TERENO preAlpine observatory (Kiese et al., 2018). With this tool we automatically calculated execution of cuts based on simulated weather depending plant development and tested the predicted timing and frequency of events with independent field data and phenological observations provided by the German Weather Service (DWD). Finally, we ran simulations of grassland biomass production spanning 2011−2100 under climate change conditions that reflect the Representative Concentration Pathways (RCP) 4.5 and 8.5, and evaluated differences in yields with dynamic and fixed schedules of management events. To further explore potentials of the dynamically adapted management under climate change conditions we conducted simulations with common nitrogen fertilization rates (200−240 kg N ha^−1^ yr^−1^) and a scenario with reduced rates (≤ 170 kg N ha^−1^ yr^−1^) following adoptions of the German fertilizer ordinance in 2018. Our hypothesis is that pre-alpine grassland simulations with static management can lead to significantly lower yields than dynamic management simulations, and that reduced rates of N fertilization will result in lower yields particularly under climate change conditions.

## 2 Material and methods

### 2.1 Study region and field site description

The new dynamic management module implemented into LandscapeDNDC (see Section 2.3) was developed, calibrated and tested with long-term field measurements of biomass harvest and respective management data of two grassland sites, i.e. Graswang and Fendt (Germany), located in the TERENO preAlpine Observatory (Kiese et al., 2018) which covers parts of the Bavarian Alps (Ammergau Mountains) and their foothills.

The high elevation site Graswang (47° 34' 12.936” N lat., 11° 1' 54.804” E lon.) is situated in an alpine valley at 864 m.a.s.l. and is characterized by a mean annual temperature (MAT) of 6.9 °C and a mean annual precipitation (MAP) of 1347 mm. The low elevation site Fendt (47° 49' 56.748” N lat., 11° 3' 39.996” E lon.) is situated in the foothills of the Alps at 595 m.a.s.l. with 8.9 °C MAT and 956 mm MAP (Table 1). The soil at Graswang is fluvic calceric Cambisol characterized by high clay as well as organic C (6.4 %) and total N (0.7 %) contents. In Fendt, a cambic Stagnosol is found with lower values of organic C (3.9 %) and total N (0.4 %) (Kiese et al., 2018).

**Table 1:** Sites used for the development, calibration and validation of the dynamic management module.

Site	Location	Altitude [m.a.s.l.]	MAT [°C]	MAP [mm]	Climate data availability/ Simulation period	Usage
Graswang	47° 34' 12.936” N lat. 11° 1' 54.804” E lon.	864	6.9	1347	2012–2018/ 2011–2100 (RCP 4.5, 8.5)	Main study sites to develop rule sets, calibrate and validate site-specific and general dynamic management module; execution of climate change scenario simulations.

Fendt	47° 49' 56.748” N lat. 11° 3' 39.996” E lon.	595	8.9	956	2012–2018/ 2011–2100 (RCP 4.5, 8.5)

Rottenbuch	47° 43' 49.152” N lat. 10° 58' 14.844” E lon.	769	8.8	1109	2012–2018	Additional study site to develop general dynamic management rule sets.

Nesselwang	47° 37' 0.12” N lat. 10° 30' 0” E lon.	870	7.43	1589	1994–2016	DWD sites with phenological observations of first cut to validate general dynamic management module.
Memmingen	47° 58' 59.88” N lat. 10° 10' 59.88” E lon.	600	8.49	964	1991–2016	

Unterhausen	47° 52' 0.12” N lat. 11° 9' 0” E lon.	550	8.47	997	1994–2016

The vegetation in Graswang is dominated by species communities of Festuca pratensis Huds., Poa pratensis L., Prunella vulgaris L., Plantago lanceolate L., Knautia arvensis (L.) J.M. Coult., Pimpinella major (L.) Huds., and Trifolium repens L, but also includes species preferring wetter conditions, like Bistorta officinalis Delarbre and Polygonum bistorta L.. Species such as Arrhenatherum elatius (L.) P. Beauv. ex J. Presl & C. Presl, Festuca rubra L., Lolium perenne L., P. lanceolata, P. vulgaris, Ranunculus repens L., T. repens, and Veronica chamaedrys L. are characteristic for the Fendt site, along with Carum carvi L., F. pratensis, Pimpinella saxifrage L., P. pratensis, and Taraxacum officinale F.H. Wigg which are dominant only at Fendt (Kiese et al., 2018).

Both grassland sites were subject to intensive management operations, equal to 4–5 cuts and 4–5 slurry applications per year following real local farmers practice in the pre-alpine study region. Mean yearly (2012−2018) yields were 10.4 ± 1.6 t DM ha^−1^ for Graswang and 11.2 ± 2.4 t DM ha^−1^ for Fendt as derived from replicated (N = 3) biomass harvests from lysimeters covering an area of 1 m². For more details on lysimeter operation see e.g. Fu et al. (2017) and Kiese et al. (2018).

### 2.2 LandscapeDNDC model overview

LandscapeDNDC is a model framework for simulating yields, water, carbon and nitrogen cycling of forest, arable and grassland ecosystems that runs with an hourly time step (Haas et al., 2013). In recent years it was successfully used and evaluated in different grassland modelling studies mainly for predicting yields, greenhouse gas emissions and nitrate leaching under current management and climate conditions (e.g. Denk et al., 2019; Houska et al., 2017; Liebermann et al., 2018, 2020; Molina-Herrera et al., 2016). LandscapeDNDC includes different sub-models for the simulation of the vegetation and the soil domain that can be combined flexibly depending on the ecosystem type and research question. The model setup of this study included the microclimate model CanopyECM (Grote et al., 2009), the hydrology model WatercycleDNDC (Kiese et al., 2011), the vegetation model PlaMo^x^ (Kraus et al., 2016; Liebermann et al., 2020) and the soil biogeochemical model MeTr^x^ (Kraus et al., 2015). All sub-models abstract the respective ecosystem domain as a vertical 1-D column assuming laterally homogeneous conditions. The following paragraphs describe the major process implementations of the individual sub-models, particularly for the model PlaMo^x^ that mainly interacts with the newly developed dynamic management model.

#### 2.2.1 CanopyECM

CanopyECM calculates the distribution of the radiation and air temperature within the canopy as well as soil temperature (Grote et al., 2009). The radiation distribution serves as input for the vegetation model in order to calculate photosynthesis, while soil temperature is essential for microbial activity in the biogeochemical soil model.

#### 2.2.2 WatercycleDNDC

WatercycleDNDC calculates the complete ecosystem water balance including throughfall and interception, evapotranspiration as well as percolation. For potential evapotranspiration, the approach of Priestley and Taylor (1972) based on the Penman-Monteith equation (Monteith, 1965) is used. Water demand for transpiration is calculated from gross photosynthesis, which is provided by the vegetation model scaled by species-specific water-use efficiency. Soil water percolation is calculated by a tipping bucket approach (Kiese et al., 2011). The simulated soil water content serves as input for the vegetation model for the determination of, e.g., drought stress and stomatal conductance as well as by the soil biogeochemical model for the determination of, e.g., microbial activity and soil diffusivity.

#### 2.2.3 PlaMo^x^

PlaMo^x^ (Fig. S1) is a general plant physiology model for different types of crops and grass species that runs on top of a photosynthesis model after Farquhar et al. (1980) and Ball et al. (1987). All simulated plant species essentially share an identical process description and are solely distinguished by species-specific parameters (Table S2), in the following labeled by ${\text{Ω}}_{x}$. PlaMo^x^ distinguishes the four plant compartments leaf, stem, roots and storage. Leaves and stems represent aboveground plant tissue directly promoting growth and structure. Storage represents an empirical bulk compartment of all compounds that do not directly support growth and structure at a given time but can be mobilized e.g., during regrowth after cutting and in spring (Chapin et al., 1990). The allocation fraction ${\text{θ}}_{x}\;$ that determines the assimilation of CO_2_ to the different plant compartments *x* is dynamic, depending on species-specific allocation parameters for the different plant compartments (${\text{Ω}}_{x}$ with x ∈{storage, root, leaf, stem}) and on the plant development state (DVS, Eq. (2)). Allocation parameters (${\text{Ω}}_{x}$) determine the compartment partition that is targeted by the plant at a given time and may deviate from the actual allocation fraction (${\text{θ}}_{x}$), e.g., after cutting events the root/shoot ratio is no more corresponding to the target partition defined by ${\text{Ω}}_{x}$ leading to an increase of ${\text{θ}}_{leaf}$ and at the same time decrease of ${\text{θ}}_{root}$ (Crider, 1955). The fraction of assimilated CO_2_ into storage increases with seasonal plant development from vegetative to reproductive growth (Eq. (1)) in order to promote initial plant growth in spring (Moore and Moser, 1995; Schulze, 1982):

(1) $${\text{θ}}_{storage}=DVS\;\times \;{\text{Ω}}_{STORAGE}$$

whereby plant development is given by accumulated growing degree days ΔGDD (Eq. (3)) and the species-specific parameter ${\text{Ω}}_{GDD}$ and ${\text{Ω}}_{T,BASE\;}$ representing total accumulated growing degree days for complete plant development and base temperature for the increment of ΔGDD, respectively:

(2) $$DVS=min\left(\;\frac{\Delta GDD}{{\text{Ω}}_{GDD}},\;1.0\right)\;$$

(3) $$\Delta GDD=\underset{}{\sum }\left(T-\;{\text{Ω}}_{T,BASE}\right)\;\;\;\;$$

The allocation of assimilated CO_2_ into roots (Eq. (4)) is given by:

(4) $${\text{θ}}_{root}=\left(1-{\text{θ}}_{storage}\right)\;\times \;\frac{{\text{Ω}}_{ROOT}\;\times \;\gamma_{cut}\;}{{\text{Ω}}_{ROOT}\;\times \;\gamma_{cut}+\;{\text{Ω}}_{LEAF}+\;{\text{Ω}}_{STEM}}\;$$

where the parameter $\gamma_{cut}$ (Eq. (5)) increases the allocation to aboveground biomass before the first cut event following the concept of the PROGRASS model (Lazzarotto et al., 2009).

(5) $$\gamma_{cut}=\left\{\begin{array}{c} {\text{Ω}}_{CUT}\;,\;first\;cut\;event\\ 1\;,\;after\;first\;cut\;event \end{array}\right)\;$$

The share of the remaining assimilated carbon between leaf and stem compartment (Eq. (6)) is determined fulfilling the following condition between actual compartment biomass *m_x_* and species-specific allocation parameters:

(6) $$\frac{m_{stem}\;}{m_{leaf}+\;m_{stem}}\;=\frac{{\text{Ω}}_{STEM}\;}{{\text{Ω}}_{LEAF}+\;{\text{Ω}}_{STEM}}\;$$

Carbon that has been allocated to the storage is translocated to other plant organs after defoliation events, e.g., cutting or grazing and at the onset of the vegetation period. At such events, all carbon from the storage is distributed according to current allocation factors.

In contrast to carbon, nitrogen is always instantaneously redistributed according to the demands from the different plant compartments. The demand of each plant compartment is given by the current dry matter biomass and optimum nitrogen concentrations ${\text{Ω}}_{NC,x}$ (x ∈{storage, root, leaf, stem}), which are assumed to be constant over time. Total plant nitrogen demand (*N_demand_*) at each time step is then given by (Eq. (7)):

(7) $$N_{demand}\;=\underset{x}{\sum }m_{x\;}\times {\text{Ω}}_{NC,x},\;\text{x}\;\in \left\{storage,\;root,\;leaf,\;stem\right\}\;$$

Leaf biomass and a species-specific parameter describing specific leaf area (${\text{Ω}}_{SLA}$) determine the leaf area index that is needed by the Farquhar and Ball based calculation of photosynthesis. Photosynthesis is further regulated by the activity of the Rubisco enzyme ($a_{rubisco}$) (Eq. (8)):

(8) $$a_{rubisco}=\;{\text{Ω}}_{RUBISCO}\times \;f_{p,drought}\times \;f_{p,temp}\times \;f_{p,nitrogen}\;$$

with the species-specific maximum rubisco activity ${\text{Ω}}_{RUBISCO}$ and the response functions $f_{p,x}$ representing the influence of drought (Eq. (9)), temperature (Eq. (10)) and nitrogen (Eq. (11)) on photosynthesis, respectively:

(9) $$f_{p,drought}=\{\begin{array}{c} min\left(1,\;\frac{\psi -\psi_{wilt}}{{\text{Ω}}_{H_{2}O}\times \;\psi_{field}-\psi_{wilt}}\right),\;\psi >\psi_{wilt}\\ 0,\;\psi \leq \psi_{wilt} \end{array}\;$$

with the soil water content ψ, the wilting point $\psi_{wilt}$, the field capacity $\psi_{field}$ and species-specific drought stress factor ${\text{Ω}}_{H_{2}O}$,

(10) $$f_{p,temp}=\{\begin{array}{c} max\left(1,\;\frac{T-0.8\;{\text{Ω}}_{LIMIT}\;}{0.2\;{\text{Ω}}_{LIMIT}}\right),\;T<{\text{Ω}}_{LIMIT}\\ 1,\;T\geq {\text{Ω}}_{LIMIT} \end{array}\;$$

with hourly resolved air temperature *T* and a species-specific critical temperature ${\text{Ω}}_{LIMIT}$ below which photosynthesis is inhibited,

(11) $$\;f_{p,nitrogen}={\frac{c_{N,LEAF}}{{\text{Ω}}_{NC,LEAF}}}^{{\text{Ω}}_{NDEF,LEAF}}\;\;\;\;$$

with the ratio of actual ($c_{N,LEAF}$) and optimum leaf nitrogen concentration ${\text{Ω}}_{NC,LEAF}$ of leafs and an exponent describing the reduction of rubisco activity under nitrogen limitation (${\text{Ω}}_{NDEF,LEAF}$).

Assimilated carbon via photosynthesis is partly metabolized by growth and maintenance respiration. Growth respiration $R_{g}\;$ (Eq. (12)) is given by fixed factor $\;({\text{Ω}}_{YIELD})$ depending on gross primary productivity (*GPP*), which is provided by the photosynthesis model after Farquhar et al. (1980) and Ball et al. (1987) that runs on top of PlaMo^x^:

(12) $$R_{g}=\;{\text{Ω}}_{YIELD}\;\times \;GPP\;$$

Growth respiration is assigned to the specific compartments depending on the current biomass allocation fraction ${\text{θ}}_{x}.$ Maintenance respiration (Eq. (13)) for all plant compartments x ∈{storage, root, leaf, stem} is given by the compartment-specific biomass $m_{x}$ and a respective maintenance respiration coefficient (Amthor, 2000):

(13) $$R_{m.x}=\;m_{x}\times \;{\text{Ω}}_{R,x}\;\times \;f_{temp}\times \;2^{\frac{T-\;{\text{Ω}}_{T,REF}}{10}}\;$$

with the same response function for low temperature as for photosynthesis and a general Q_10_ temperature dependency with increasing temperature.

Non-respiratory plant carbon losses include root exudation and plant senescence. Root exudation is given as a fraction related to root respiration (Eq. (14)):

(14) $$R_{m.x}=\;{\text{Ω}}_{EXUDATE}\times \;{\text{R}}_{g,root}\;$$

Plant senescence (Eq. (15)) is given by the maximum of a set of response functions $f_{s,x}$ with regard to drought (Eq. (16)), frost (Eq. (17)) and plant age (Eq. (18)):

(15) $$S_{x}=\max\left(f_{s,drought,}\;\;f_{s,frost},\;\;f_{s,age}\right)\times \;m_{x}\;$$

with x ∈{storage, root, leaf, stem}.

These response functions are:

(16) $$f_{s,drought}=\;\{\begin{array}{c} {\text{Ω}}_{SEN,DROUGHT}\times \left(1-\;min\left(1,\;\frac{\psi -\psi_{wilt}}{{\text{Ω}}_{H_{2}O,SEN}\times \;\psi_{field}-\psi_{wilt}}\right)\right),\;\psi >\psi_{wilt}\\ 0,\;\psi \leq \psi_{wilt} \end{array}$$

in which the species-specific drought stress factor ${\text{Ω}}_{H_{2}O,SEN}$ is similarly defined as compared to the drought influence on photosynthesis,

(17) $$f_{s,frost}=\{\begin{array}{c} {\text{Ω}}_{SEN,FROST}\;\times \;\left|T\right|,\;T<0\\ \;\;0,\;T\geq 0 \end{array}\;$$

with the hourly resolved temperature *T* in the air and the soil for above- and belowground senescence, respectively.

(18) $$f_{s,age}=\;{\text{Ω}}_{SEN,AGE}\;$$

#### 2.2.4 MeTr^x^

The MeTr^x^ model simulates soil carbon and nitrogen turnover and the associated processes humification, mineralization, nitrification, denitrification and ammonia volatilisation (Kraus et al., 2015). These processes are key for the simulation of inorganic nitrogen substrate availability (NH_4_, NO_3_) for plant uptake and microbial driven production and emissions of C (CO_2_) and N (NO, N_2_O, N_2_) emissions as well as other loses such as NO_3_ leaching and NH_3_ emissions. In addition to substrate availability (usually in form of Michaelis-Menten kinetics), all microbial processes depend on soil moisture and soil temperature, which are provided by above-described sub-models as well as the model input quantities pH and soil texture.

### 2.3 Dynamic management module

For grassland simulations, the LandscapeDNDC management module requires inputs for execution of cutting and manuring events and further information on quantity and composition of the applied manure (see Section 2.4), which all were previously read from a user derived management input file.

#### 2.3.1 Description

**Fig. 1:**
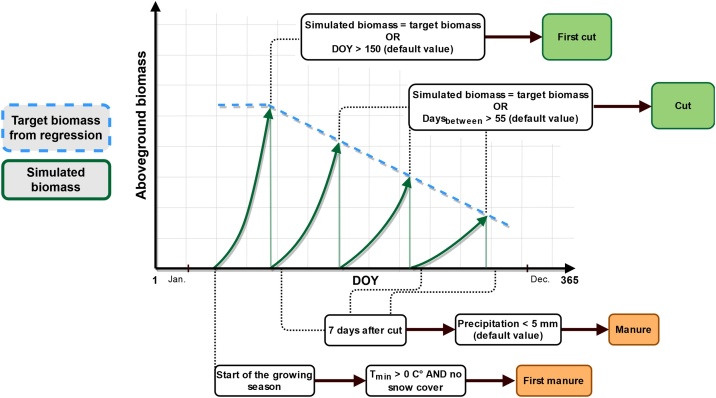
Scheme of rule sets of the dynamic management model; “default value” highlights a parameter which can be changed with the input file; DOY = day of the year; Days_between_ = maximum count of days between two cuts; T_min_ = daily minimum temperature (C°); start of the growing season as simulated by the vegetation sub-model.

The dynamic management model was developed from long term field data (2012–2016) of a total of 22 biomass harvests (N = 3) (kg DM ha^−1^) and respective cutting dates (DOY, day of the year) following actual farmers’ practice in the study region. These data were used to fit a linear regression to maximum standing biomass versus time, which allows to define a “target biomass” for executing a cutting event for any DOY. Hence, in the dynamic management model a cut is scheduled if the target biomass at a given DOY exceeds the threshold given by the regression equation (Fig. 1).

To calculate the target biomass for each cutting event we differentiated between a site-specific regression approach (Graswang r² = 0.39, p < 0.001; Fendt r² = 0.57, p < 0.001) directly derived from field data (target biomass=m*DOY+b) and a general approach that can be applied for intensive grasslands in the pre-alpine study region in the absence of detailed yield data (app. 500−1000 m.a.s.l.). For the latter, in addition to biomass harvest data of Graswang (864 m.a.s.l.) and Fendt (595 m.a.s.l.) we also used further data of the TERENO site Rottenbuch (769 m.a.s.l.; 47° 43′ 49.152′' N lat., 10° 58′ 14.844′' E lon., Table 1). We calculated the relative contribution (in %) of each cutting event to the annual biomass production which continuously decreased with number of cuts (r² = 0.55, p < .0001; see Fig. S2). These relative contributions can be translated into biomass thresholds by multiplying them with the expected annual biomass production of a given grassland site, which is set as an additional input parameter for the dynamic management model of LandscapeDNDC. This value can either be derived from available measurements or alternatively from a regression model estimating annual yields (AGB in dt dry matter ha^−1^ yr^−1^) of intensively (4–5 cuts) used grasslands depending on elevation (h in m.a.s.l.) (Eq. (19)) as derived from managed grassland systems in Switzerland (Richner and Sinaj, 2017).

(19) AGB=159−0.058*h 

We compared results from this function considering respective elevations of the three study sites Graswang, Rottenbuch and Fendt and found only minor deviations of −1.8 % to −7.1 % from the field measurements.

If the target biomass is not reached after a given time (day of the year: DOY), further rules are considered (see also Fig. 1), which also evolve from field data and reflect farmer’s decision-making under unfavorable grassland growth conditions such as drought or cold spring:

1.) If the target biomass of the first cutting event is not reached after DOY 150, the first cut will be set at DOY 151.
2.) If the target biomass for all following cutting events is not reached within 55 days, they will be set at DOY 56 after the previous cutting event.

Since timing of manure events is highly related to timing of cutting events, we defined the following rules regarding manure applications:

1.) The first manure event is scheduled at the start of the growing season as simulated by the vegetation sub-model but only at times without snow cover or frozen soil. Due to national legislation (Achilles et al., 2018) manure events in any case are not scheduled before the 1^st^ of February.
2.) All other manure applications are scheduled within 7 days after respective cutting events on the first day without heavy rain (< 5 mm). Note that due to regional farmers practice and according to recommendations from extension services no manure is applied after the second cut. In line with legislation driven limitation of fertilization rates to ≤ 170 kg N ha^−1^ yr^−1^ (Achilles et al., 2018) for the reduced nitrogen scenario, manure events are scheduled only before the first cutting and after the first and the third cutting event.

#### 2.3.2 Calibration and validation

First, we examined the capability of the site-specific and the general regression model to reproduce the field data management at Graswang and Fendt. To do so we split the available data into a calibration (2012–2014) and a validation (2015–2018) period and ran simulations with weather data from on-site climate stations (see Section 2.4 for a detailed description of climate, soil and vegetation model inputs).

We further tested the dynamic management module for its capability to simulate the timing of the first cut and the start of the growing season as given by phenological data routinely recorded by the German Weather Service (DWD, Kaspar et al., 2014). Observations from 59 DWD sites were available regarding the day of greening (equal to the start of the growing season) i.e. 25 % of the grassland canopy characterized by fresh green leaves, while data from 53 DWD sites were available regarding the day of first cut in the Bavarian pre-alpine study region (48.05–47.56 latitude and 12.60–10.02 longitude and 500–1000 m.a.s.l.) between 1991 and 2016.

For more detailed testing of the general regression approach, we compared the simulated first cut and start of the growing season with observations of three phenological DWD sites representing different elevation levels (Table 1). Further selection criteria were completeness of phenological time series and availability of weather data from nearby DWD climate stations. Eventually, the following three sites were selected: 1) phenological site Nesselwang (47° 37′ 0.12′' N lat., 10° 30′ 0′' E lon., 870 m.a.s.l.) with DWD climate station Oy-Mittelberg (8.56 km distance, 47° 38′ 10.32′' N lat., 10° 23′ 21.12′' E lon., 885 m.a.s.l., 7.43 °C MAT, 1589 mm MAP), 2) phenological site Memmingen (47° 58′ 59.88′' N lat., 10° 10′ 59.88′' E lon., 600 m.a.s.l.) with DWD climate station Memmingen (3.34 km distance, 47° 58′ 55.2′' N lat., 10° 8′ 18.24′' E lon., 615 m.a.s.l., 8.49 °C MAT, 964 mm MAP), 3) phenological site Unterhausen (47° 52′ 0.12′' N lat., 11° 9′ 0′' E lon., 550 m.a.s.l.) with DWD climate station Raisting (5.73 km distance, 47° 54′ 32.76′' N lat., 11° 6′ 17.28′' E lon., 553 m.a.s.l. from 01.01.1994 to 31.01.1999, with 8.2 °C MAT and 1007 mm MAP) and with DWD station Wielenbach (1.92 km distance, 47° 52′ 57.72′' N lat., 11° 9′ 27.36′' E lon., 550 m.a.s.l. from 01.02.1999 to 31.01.2016, with 8.74 °C MAT and 987 mm MAP).

Since no detailed soil input for these sites were available we initialized all three sites with soil characteristics of the Graswang site (see Section 2.4). For derivation of the average yearly biomass, we used the formula for intensively managed grasslands described in Section 2.3.1.

### 2.4 LandscapeDNDC model simulations

The simulated development of aboveground biomass, soil carbon and nitrogen dynamics depend on soil characteristics (Table 2), vegetation growth parameters (Table S2), weather conditions as well as field management operations. Soil organic carbon and nitrogen is described by various empirical pool quantities representing different age and decomposition classes. During a spin-up time of two years, pools of soil organic matter are brought into equilibrium with prevailing management, soil and climate conditions.

**Table 2:** Physical and chemical top soil (0–10 cm) characteristics of the grassland sites Fendt and Graswang; BD = bulk density, C_org_ = organic carbon content, N_org_ = organic nitrogen content, FC = field capacity, PWP = permanent wilting point, HC = hydraulic conductivity.

Sites	Graswang		Fendt	
Depths	0 – 5	5 – 10	0 – 5	5 – 10
BD [g kg^−1^]	0.552	0.82	0.74	1.1
pH	4.9	7.1	5.1	6.6
C_org_ [Weight-%]	10.02	5.81	6.79	4.35
N_org_ [Weight-%]	1.001	0.67	0.66	0.48
Clay fraction [%]	58.5	58.5	27.2	25.2
Silt fraction [%]	35.1	35.1	40.3	40.3
Sand fraction [%]	6.4	6.4	32.5	34.5
FC (pF 1.8) [Vol.-%]	52.0	52.0	50.0	46.0
PWP (pF 4.2) [Vol.-%]	22.1	22.1	23.5	23.5
HC [cm min^−1^]	0.005	0.005	0.020	0.020
Stone fraction [%]	1.0	1.5	0.0	3.8

#### 2.4.1 Grassland management and simulations

As mentioned in Section 2.3 management input requires in addition to dates further information on quantity and composition of the applied manure. This includes the pH value, the total amounts of carbon (kg C ha^−1^), the C:N ratio and if available information on the partitioning of nitrogen in fractions of NH_4_^+^, NO_3_, UREA and dissolved organic nitrogen (DON). For our study, information on cutting and manuring dates and quantities were available for the time period 2012−2018. Slurry composition was derived from analysis of slurry samples (N = 19; Raiffeisen Laborservice, Ormont, Germany) of each fertilization event from 2012 to 2016. Mean slurry carbon and nitrogen loads and pH values were 437 ± 130 kg C ha^−1^ and 48 ± 10 kg N ha^−1^ and 7.6 ± 0.4, respectively. Given this information on grassland management, which is often not available in this detail (Kipling et al., 2016; Luostarinen et al., 2018), we conducted the following numerical experiments:

i) for an overall evaluation of LandscapeDNDC grassland biomass predictions (2012–2018) we used real time dates of single cutting and manuring events and measurements of manure composition (with annual fertilization rates ranging between 182 and 248 kg N ha^−1^ yr^−1^);
ii) for climate change scenario simulations (2011–2100) with static management settings we used mean cutting and manuring dates of 2012–2018 (i.e. 4 cuts and 4 manure events, the latter equal to 192 kg N ha^−1^ yr^−1^);
iii) for climate change scenario simulations (2011–2100) with dynamic management we derived cutting and manure events on the fly of simulations with the dynamic management module for a scenario with previously common fertilization rates (200−240 kg N ha^−1^ yr^−1^) and a scenario with reduced nitrogen fertilization (≤ 170 kg N ha^−1^ yr^−1^) following changes in legislation in 2018 (see also Section 2.3, Achilles et al., 2018).

Note that for ii) and iii) manure characteristics were represented by means of measurements of 2012 to 2016. For the limited nitrogen scenario only, we slightly adjusted total carbon and nitrogen loads per event to achieve a maximum of 170 kg N ha^−1^ yr^−1^.

#### 2.4.2 Soil and vegetation

LandscapeDNDC allows a flexible vertical parameterization of the soil profile, depending on available measurements. Table 2 provides essential soil input of LandscapeDNDC for the two simulated sites Graswang and Fendt exemplarily for the top soil. In addition to data provided in Table 2, for our simulations we used further soil profile information of up to ten soil horizons down to 140 cm soil depth (Kiese et al., 2018; see Table S1).

LandscapeDNDC was mainly developed and validated for single species setups (mainly crops in arable systems) rather than for simulating complex plant communities e.g. characterized by multiple plant functional types, a main feature of many grassland ecosystems. Therefore, we simulated grass growth still by the single species approach but in our case growth parameters represent mean values (see Table S2) which originate from the calibration to the plant mixtures (see Section 2.1) occurring at the two investigated grassland sites.

#### 2.4.3 Weather data and climate change scenarios

LandscapeDNDC uses hourly or daily information on precipitation [mm], minimum and maximum air temperature [°C] and global radiation [W m^−^²], which were available from weather stations operating since 2012 at the two study sites Fendt and Graswang. In case of daily time resolution LandscapeDNDC uses well-established algorithms to convert data in hourly time resolution (Berninger, 1994; Chow and Levermore, 2007).

Due to substantial biases in dynamically regionalized global climate models, particularly for precipitation in complex alpine terrains (Smiatek et al., 2016), site specific daily climate change scenarios (RCP 4.5 and 8.5) for the time period of 2011–2100 were developed with the stochastic weather generator LARSWG (Semenov and Barrow, 1997; Semenov and Stratonovitch, 2010) which is a widely used tool in crop modelling studies (e.g. De Bruijn et al., 2012; Lazzarotto et al., 2010). LARSWG generates daily climate series of precipitation, global radiation and minimum and maximum air temperature based on probability distributions and correlations of long-term observed weather variables at intended sites. Climate projections from global climate models (GCM) are used to calculate climatic changes for a given site that are applied on these parameter distributions to create site specific climate change scenario series (Semenov and Stratonovitch, 2010). To do so, LARSWG can make use of CMIP5 (Coupled Model Intercomparison Project Phase 5) global climate projections (Taylor et al., 2012) from which we selected output of HadGEM2-ES, since it was shown to represent the height- and latitude-dependent temperature and precipitation pattern over the alpine region reasonably well (Zubler et al., 2016). In order to assess the statistical uncertainty of the generated climate time series, LARSWG was used to generate ten different realizations for each site.

Since climate stations in Graswang and Fendt have only been operated since 2012, LARSWG calculations were informed instead by weather data from longer observation records of nearby stations of the German Weather Service (DWD). For Fendt, precipitation and air temperature data were taken from 17 years (2000–2017) time series of the DWD station Wielenbach (47° 53′ 2.4′' N lat., 11° 9′ 28.8′' E lon., 545 m.a.s.l., 9.16 km distance) with a MAP of 968 mm (Fendt site: 956 mm), minimum MAT of 3.56 °C (Fendt site: 3.54 °C) and a maximum MAT of 14.68 °C (Fendt site: 14.32 °C). For Graswang, precipitation was derived from a 15 years time series (2002–2017) of the DWD rainfall station Ettal-Graswang (47° 34′ 19.2′' N lat., 11° 1′ 26.4′' E lon., 872 m.a.s.l., 619.3 m distance) with a MAP of 1545 mm which is reasonable higher (+ 198 mm) than MAP measured on site. For the Graswang site air temperature was taken from the DWD station Garmisch-Partenkirchen (47° 28′ 58.8′' N lat., 11° 3′ 43.2′' E lon., 719 m.a.s.l., 9.97 km distance) but due to systematic differences in MAT (minimum MAT 2.65 °C, maximum MAT 13.94 °C) these data were corrected using a linear regression of Graswang and Garmisch air temperature data for the years 2012 to 2016: T_MAX Graswang_ = 0.9359 * T_MAX Garmisch_ – 0.915 (r² = 0.88) and T_MIN Graswang_ = 0.993 * T_MIN Garmisch_ – 1.3523 (r² = 0.91).

Global radiation was taken for both sites from DWD station Hohenpeißenberg (47° 48′ 3.24′' N lat., 11° 0′ 38.88′' E lon., 977 m.a.s.l., 25.8 km distance to Graswang, 5.24 km distance to Fendt).

Within the RCP 4.5 scenario a mean annual temperature increase within the vegetation period of maximum 1.4 °C is predicted from 2011 to 2070 and from thereon less steep by up to 1.7 °C in the year 2100 (Table 3). For RCP 8.5 a continuous temperature increase of 1.9 °C (Graswang and Fendt) until 2070 and of up to 4.4 °C in 2100 are reported. The mean annual precipitation for both sites for RCP 4.5 is slightly decreasing towards 2070 with a tendency to increase again after 2070 until 2100. For RCP 8.5 the precipitation further decreases after 2070 which results in overall 107−172 mm less annual precipitation at the end of the simulation period compared to the first period between 2011–2040.

**Table 3:** Average climatic conditions (± SD) in the vegetation period (March to October) of the two sites Graswang and Fendt originating from 10 realizations of site specific climate change scenarios generated by LARSWG and based on the HadGEM2-ES climate projection over 30-year periods from 2011 to 2100. T = temperature in °C; PREC = precipitation in mm.

Site	RCP	Period	T [°C]	PREC [mm]
Graswang	4.5	2011–2040	10.8 ± 0.5	1219 ± 148
		2041–2070	12.1 ± 0.4	1165 ± 167
		2071–2100	12.5 ± 0.3	1193 ± 168
	8.5	2011–2040	10.8 ± 0.5	1245 ± 161
		2041–2070	12.7 ± 0.6	1175 ± 156
		2071–2100	15.2 ± 0.4	1073 ± 153
Fendt	4.5	2011–2040	13.2 ± 0.4	757 ± 117
		2041–2070	14.6 ± 0.4	712 ± 119
		2071–2100	14.9 ± 0.2	752 ± 112
	8.5	2011–2040	13.2 ± 0.5	778 ± 111
		2041–2070	15.1 ± 0.6	741 ± 124
		2071–2100	17.6 ± 0.4	671 ± 109

For all simulations under current climate conditions we set atmospheric CO_2_ concentrations to a fixed value of 400 ppm, while transiently (yearly) increasing atmospheric CO_2_ concentrations were used for the climate change scenarios based on the datasets provided by Meinshausen et al. (2011), reaching maximum values of 538 ppm and 936 ppm CO_2_ in 2100 in RCP 4.5 and RCP 8.5 scenarios, respectively.

### 2.5 Statistical analysis

To evaluate model performance on biomass production, dynamically simulated cutting dates and start of the growing season as well as to analyze trends in the DWD phenological datasets, we used linear regression models and respective coefficients of determination (r^2^) as well as the concordance correlation coefficient (CCC) (Lin, 1989). Root mean square errors (RMSE) and normalized root mean square errors (NRMSE, =RMSE/average of observed values) were calculated to account for differences between observed and simulated aboveground biomass harvests for the period of 2012−2018. Additionally, for cutting dates in this reference period a paired *t*-test on the group mean values of measured and simulated values was conducted (α = 0.05). To describe changes in biomass harvest variability between years with climate change, we calculated coefficients of variation for the periods 2011−2040 and 2071−2100 (CV = standard deviation / arithmetic mean).

For tests on normality of the empirical distribution for any parameter, we used the Shapiro-Wilk test. In case of normal distributed data, we assessed correlation using the Pearson correlation coefficient. For non-normally distributed data, the Spearman rank test was used.

All statistical analysis and figures were generated using SAS/STAT software, Version 9.4 of the SAS System for Windows. Copyright © 2012−2018 SAS Institute Inc. SAS and all other SAS Institute Inc. product or service names are registered trademarks or trademarks of SAS Institute Inc., Cary, NC, USA.

## 3 Results

### 3.1 Aboveground biomass simulations

**Fig. 2:**
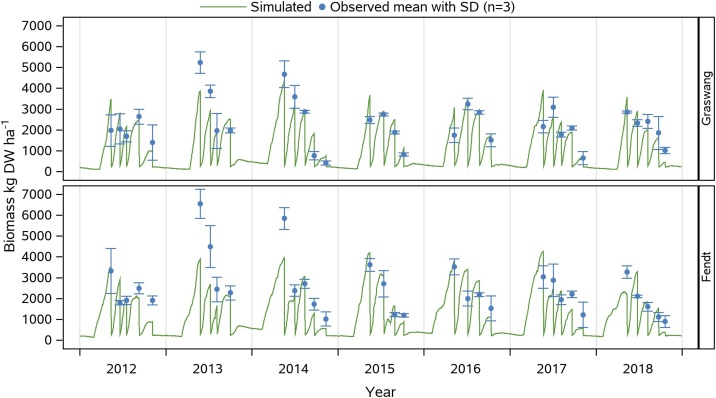
Simulated and mean ± SD measured (n = 3) aboveground biomass (in kg DW ha^−1^) during 2012 to 2018 at the two grassland sites Graswang (top) and Fendt (bottom).

Robust simulations of grassland biomass development and yields at respective cutting events are essential for the applicability of LandscapeDNDC for evaluation of grassland functions under current and climate change conditions. Fig. 2 shows the temporal development of simulated and measured harvested biomass (n = 3) at cutting events (2012−2018) of LandsapeDNDC being parametrized with specific climate, soil and management information for the Fendt and Graswang sites.

Disregarding the year 2013 with exceptional high measured biomass for the first two cuts in Graswang and Fendt and the first cutting event in Fendt in 2014, patterns and magnitude of the simulated biomasses were mostly consistent with measurements. Statistical measures of the calibration (2012–2014) and validation (2015–2018) period were in the same range (Graswang: r² = 0.62−0.71, p < .0001; Fendt: r² = 0.64−0.66, p < .0001) with the only exception in the calibration period for Fendt with a higher RMSE value of 1127 kg DW ha^−1^ (NRMSE = 38.6 %). Considering the complete simulation period of seven years with a total of 32 cutting events resulted in RMSE of 720 and 917 kg DW ha^−1^ and r² of 0.61 (p < .0001) and 0.52 (p < .0001) for Graswang and Fendt, respectively (NRMSE: Graswang = 31.7 %, Fendt = 37.1 %).

### 3.2 Dynamic management simulations

**Fig. 3:**
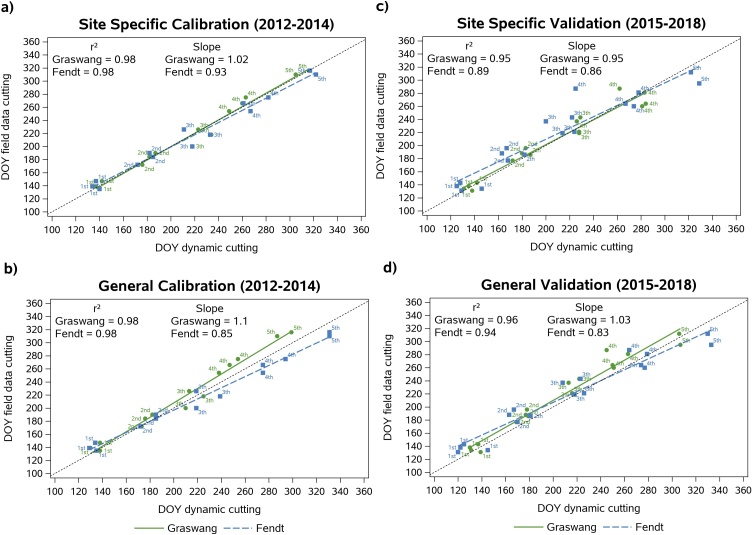
Correlation of dynamically simulated and observed Day of Year (DOY) of cutting events for the calibration (2012 to 2014; a and b) and the validation (2015 to 2018; c and d) period with the site-specific (a and c) and the general regression approach (b and d), 1st = first cut; 2nd = second cut etc.

Fig. 3 shows the comparison of dynamically simulated and observed cutting DOY for the calibration period 2012–2014 and the validation period 2015−2018. For both periods, the dynamic simulations accurately represented the timing of cutting events (r² = 0.89−0.98). The performance of the general approach was only slightly lower than the performance of the site-specific approach, with a tendency in the calibration period towards later simulated cuts for the warmer Fendt site and earlier simulated cuts for the colder Graswang site after the third cut. This also shows up by higher deviations of the slope, with values < 1 at Fendt and > 1 at Graswang, respectively. Group means of the cutting DOY at 1^st^ to 5^th^ cuts were not significantly different from measured values (*t*-test; p > 0.05) but due to error propagation deviations of simulations and field measurements increased with increasing number of cuts (Fig. 3).

At both sites, the simulated number of yearly cuts and the total number of cuts during the full 7-year simulation period match very well with field observations (Table 4). Simulated counts of cutting events per year deviate by a maximum of ± 1 from observed data. Regarding all 32 cutting events, both site-specific and general simulations slightly overestimated the number of cutting events by a maximum of three cuts.

**Table 4:** Deviations of cutting events between simulations and observations during the period 2012 to 2018.

Year	2012	2013	2014	2015	2016	2017	2018	2012–2018
Field data	5	4	5	4	4	5	5	32
Graswang site-specific	–	+1	–	–	+1	−1	−1	–
Fendt site-specific	–	–	–	+1	+1	–	–	+2
Graswang general	–	+1	–	+1	+1	–	–	+3
Fendt general	–	–	–	–	+1	–	–	+1

**Fig. 4:**
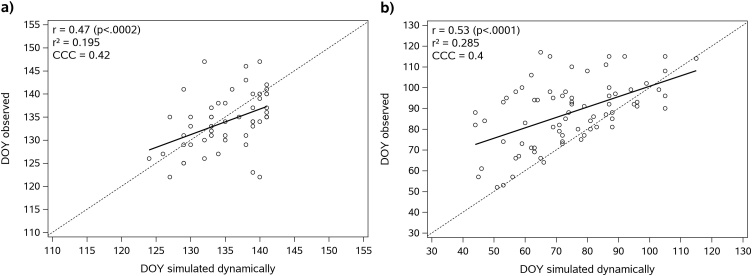
Correlation of simulated (general approach) and observed (a) first cutting events and (b) start of the growing season for phenological German Weather Service (DWD) stations Nesselwang, Memmingen and Unterhausen.

In addition to the detailed validation of predicted cutting events with TERENO field data we compared LandscapeDNDC simulations also with observations of three phenological sites of the German Weather Service (DWD), namely Nesselwang, Memmingen and Unterhausen. Fig. 4a shows the correlation between simulated (general approach) and observed first cutting events for all three sites. Despite a pronounced scattering of simulated and observed data, the correlation was significant (r = 0.47; p < 0.002). In 74 % of the cases the model predicted the first cut within ± 7 days of the observed date with a corresponding RMSE of 7.8 days. The average difference between simulated and observed cuts was 2.2 ± 7.5 days.

For the start of the growing season (Fig. 4b) a stronger correlation (r = 0.53, p < 0.001) between simulated and observed dates was found, but the mean deviation of −13.5 ± 15.7 days revealed a bias towards an earlier simulated start of the growing season as compared to observations. As a result, only 40 % of the simulated values were within ± 7 days of the observed dates, and the RMSE was also higher (20.7 days).

### 3.3 Grassland management predictions under climate change conditions

#### 3.3.1 Shift of the start of the growing season and the first cut

**Fig. 5:**
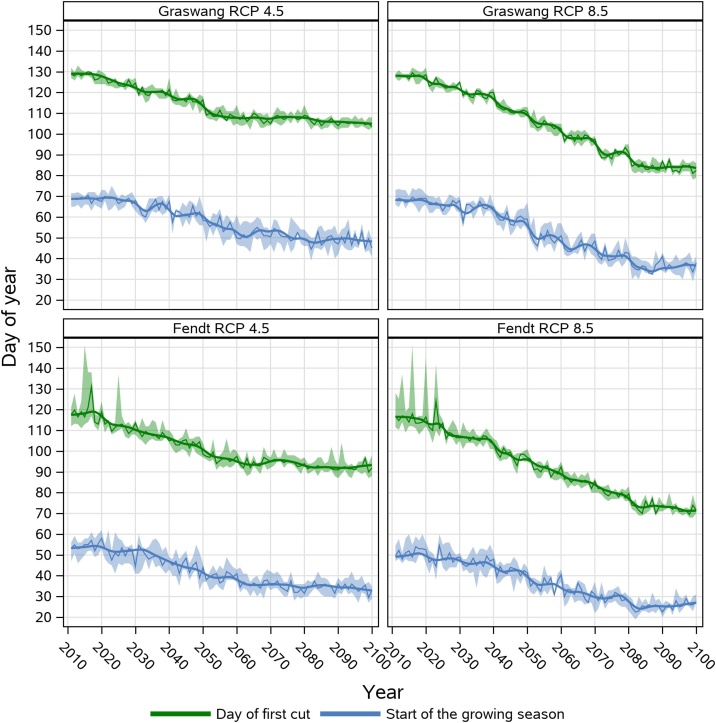
Simulated day of first cut and start of the growing season at Graswang and Fendt sites for RCP 4.5 and 8.5 emission scenarios. Shown are median with 5-year moving average (solid lines) and band of 25^th^ and 75^th^ percentiles originating from 10 realizations of site specific climate change scenarios generated by LARSWG and based on the HadGEM2-ES climate projection.

As the validation results for the reference period did not show any significant differences in model performance between the site-specific and the general dynamic management approach, we present here only data of the general approach. Fig. 5 depicts the temporal progression of the start of the growing season and the day of the first cutting event of simulations based on the RCP 4.5 and RCP 8.5 climate change scenarios for the Fendt and Graswang sites.

In both RCP scenarios with progression of time a clear trend towards an earlier simulated start of the growing season and first cutting events are evident (Fig. 5). Simulated first cutting events at the higher elevation site Graswang changed from DOY 130 to 105 in the RCP 4.5 scenario and to DOY 85 in the RCP 8.5 scenario. For the warmer site Fendt comparable temporal patterns and differences between RCP 4.5 and the RCP 8.5 were observed, however DOYs of the first cutting events were in both scenarios approximately 10 days earlier as compared to Graswang. Compared to changes in the dates of the first cutting event, at both sites, simulated changes of the start of the growing season were less early and differences of the temporal development between the RCP 4.5 and 8.5 scenarios were smaller.

#### 3.3.2 Validation of simulations against DWD phenological observations

**Fig. 6:**
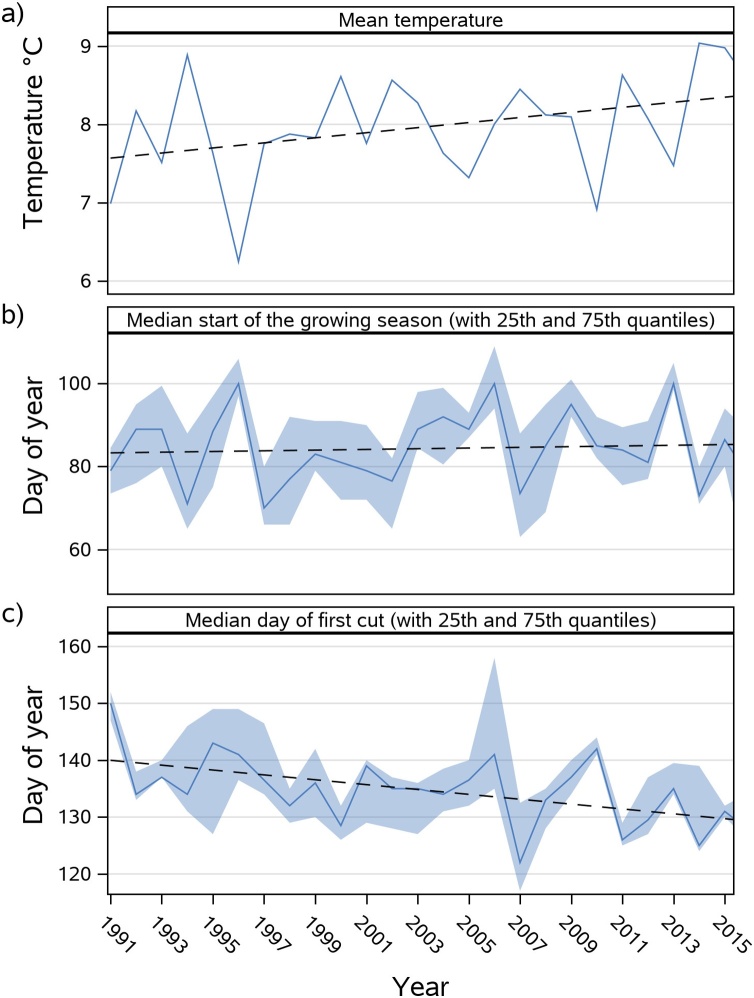
Temporal development of a) mean annual air temperature based on means of 5 stations of the German Weather Service (DWD): Nesselwang, Memmingen, Unterhausen, Kempten (705 m.a.s.l., 47° 43' 23.88” N lat., 10° 20' 5.28” E lon.) and Hohenpeißenberg (977 m.a.s.l., 47° 48' 3.24” N lat., 11° 0' 38.88” E lon.) in the pre-alpine region of Bavaria; b) start of the growing season and c) the day of the first cut, the latter two resulting from a compilation of phenological observations of the DWD in the pre-alpine region of Bavaria.

Compilation of data of the start of the growing season and first cut from >50 sites of the phenological observation network of the German Weather Service (DWD) located in the pre-Alpine study region revealed a significant trend towards earlier dates of first cuts from 1991 to 2016 (r² = 0.25, p < 0.05), following the trend of increasing mean annual air temperatures during this time period (correlation of first cutting dates and temperature; r = 0.72, p < .0001) (Fig. 6). A shift of 4.5–6.7 days (representing 25^th^ and 75^th^ percentiles) towards earlier first cuts between two periods 1991−2000 and 2007–2016 was observed. Referencing this to the mean temperature increase in the same period of +0.48 °C results in an earlier timing of the first cut between 9.4–14.0 days per 1 °C temperature increase.

Results of the RCP climate scenario simulations of LandscapeDNDC for an equally long period (2011−2040) agreed well with these observations with a similar range of 9.1–16.9 days earlier first cutting dates referenced to a temperature increase of 1 °C (Table 5).

**Table 5:** Shift of the first cut (calculated between decadal means), in days, scaled to +1 °C air temperature increase as calculated from observations of the German Weather Service (DWD) (see Fig. 6) and LandscapeDNDC climate change scenario simulations with the general dynamic management parametrization. Rates represent 25th and 75th quantiles of replicated observations and simulations.

Scenario	Shifted days per +1 °C
Graswang RCP 4.5 2011–2040	−9.1 to −9.9
Graswang RCP 8.5 2011–2040	−10.3 to −10.4
Fendt RCP 4.5 2011–2040	−10.7 to −16.8
Fendt RCP 8.5 2011–2040	−9.9 to −16.9
Phenology data from DWD 1991–2016	−9.4 to −14.0

In contrast to the shifts observed for first cutting dates, the DWD phenological observations do not show a clear trend of changes in the timing of the start of the growing season (Fig. 6) with median values spreading between DOY 70 and 100. Interestingly, and following DWD observations LandscapeDNDC RCP scenario simulations also do not show a clear trend until approximately 2030. Nevertheless, for both sites, the simulated start of the growing season is about up to 20 and 30 days earlier in 2080 and stabilize towards 2100 for the RCP 4.5 and 8.5 scenario, respectively (Fig. 5).

#### 3.3.3 Influence on number of yearly cuts

Trends towards an earlier start of the growing season and first cutting dates as simulated by the dynamic management routine of LandscapeDNDC influenced also the total number of cutting events per year. For the > 200 kg N dynamic simulations the number of cuts increased at both sites and in both RCPs from alternating between four and five cuts (2011−2035) to regularly five cuts after 2035. For the RCP 8.5 scenario from 2080 onwards, even six cuts were simulated at the warmer Fendt site and after 2090 likewise for the colder Graswang site. Within the reduced N scenarios, four cuts were constantly simulated for both sites between 2011 and 2035 and a slower increase to a maximum of five cuts thereafter. Five cuts were continuously simulated from 2045 at the earliest for Fendt RCP 8.5 and from 2080 at the latest for Graswang RCP 4.5 without a predicted increase towards six cutting events.

#### 3.3.4 Grassland biomass production under climate change conditions

The previous findings of dynamic grassland management simulations showed that climate change beside earlier execution of the first cut result in increasing number of cuts and associated manure events, features which cannot be reflected by static management or if annual fertilization rates are restricted to 170 kg N ha^−1^ as required by legislation since 2018.

**Fig. 7:**
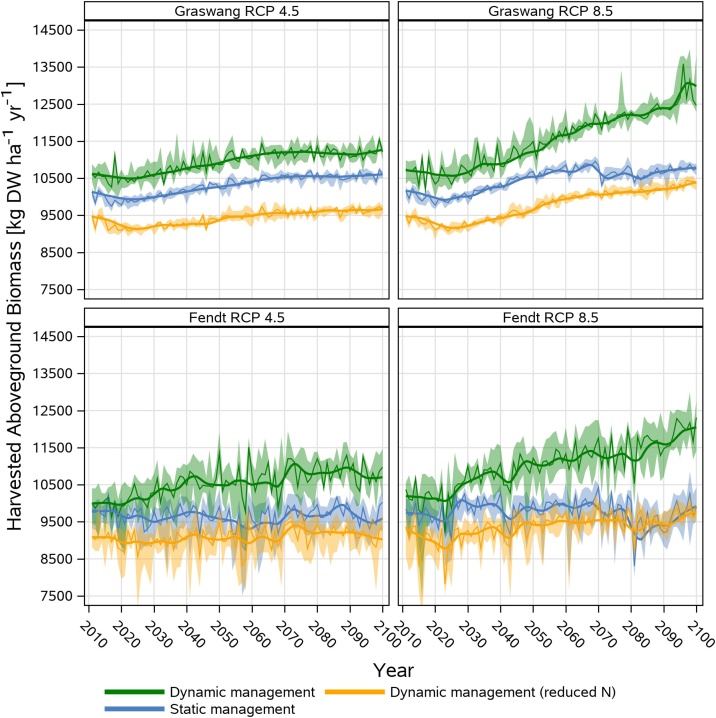
Comparison of aboveground biomass harvest (in kg DW ha^−1^ yr^−1^) for the Graswang and Fendt sites for RCP 4.5 and 8.5 scenarios as simulated by dynamic (with and without nitrogen (N) fertilization reduction) and static management. Shown are median with 5-year moving average (solid lines) and bands of 25^th^ and 75^th^ percentiles originating from 10 realizations of site specific climate change scenarios generated by LARSWG and based on the HadGEM2-ES climate projection.

For Graswang and the RCP 4.5 scenario, the dynamic reduced N scenario showed lower biomass yields of about 1000–1600 kg DW ha^−1^ yr^−1^ as compared to the higher loads of N fertilization under static and the dynamic management. Within RCP 8.5 simulations, the yield differences between the static and the reduced N management decreased in the 2071−2100 period (< 500 kg DW ha^−1^ yr^−1^) while the difference to the dynamic non-reduced N scenario increased to 2159 kg DW ha^−1^ yr^−1^. Overall, climate change induced increases of yields of the three management scenarios were about 500 kg DW ha^−1^ yr^−1^ between the period of 2011−2040 and 2071−2100 in RCP 4.5 and 8.5, except for the dynamic management and the RCP 8.5 scenario where yield increases for the same period of time with 1600 kg DW ha^−1^ yr^−1^ were much higher (Fig. 7).

As compared to Graswang lower differences (500 kg DW ha^−1^ yr^−1^) between the static and dynamic reduced N scenario were simulated for Fendt in the RCP 4.5 and RCP 8.5 scenario for the period 2011−2040 which further decreased in the period 2071−2100. In contrast to Graswang, climate change as predicted by RCP 4.5 did not lead to increasing grassland biomass under static and the dynamic reduced N management, while for the dynamic non-reduced N management increases of about 650 kg DW ha^−1^ yr^−1^ were predicted during both periods 2011−2040 and 2071−2100. In the RCP 8.5 scenario grassland yield increases under dynamic non-reduced N management at Fendt were similar to Graswang. This was not the case for the static and dynamic reduced N management which both showed even a decreasing trend from 2060 onwards. The yield increase in RCP 8.5 for the dynamic non-reduced N management resulted in a mean biomass of 11606 kg DW ha^−1^ yr^−1^ for the 2071−2100 period, which is about 1170 kg higher as compared to the start of the simulation period (2011−2040) and about 2000 kg DW ha^−1^ yr^−1^ higher than the mean biomass associated with static (9642 kg DW ha^−1^ yr^−1^) and dynamic non-reduced N (9557 kg DW ha^−1^ yr^−1^) management operations for 2071−2100.

**Fig. 8:**
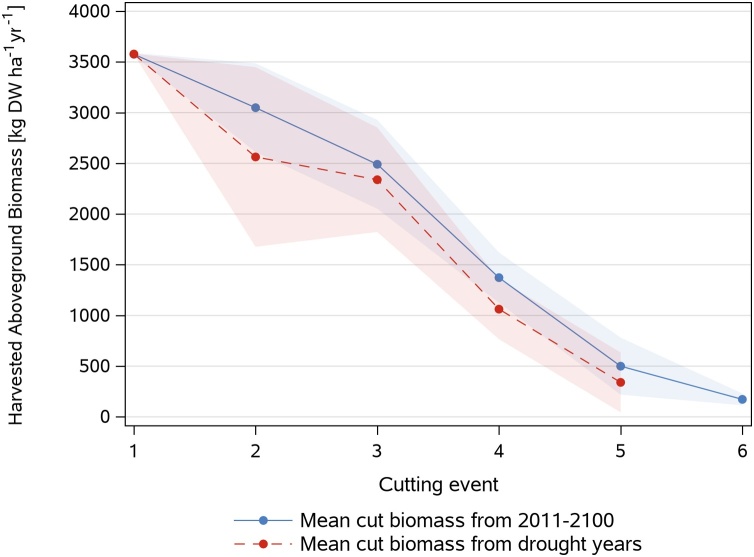
Comparison of mean +/- SD (bands) simulated biomass per cutting event of drought years (2043, 2048, 2063, 2081, 2086, 2093, 2095 and 2099; growing season precipitation < 550 mm) with mean +/- SD (bands) simulated biomass of the period 2011-2100 for the RCP 8.5 climate scenario at Fendt.

At the warmer Fendt site simulated yields showed overall higher differences across years (Fig. 7) which is also documented by higher coefficients of variation ranging between 3–5 % at Graswang and 7–10 % at the Fendt site. With regard to climate change at both sites the variability of yields were not different for the period 2011−2040 and 2071−2100 neither for RCP 4.5 nor RCP 8.5. Nevertheless, as shown in Fig. 8 yields of occasional drought years defined by < 550 mm growing season (March-October) precipitation were about 15 % lower than in non-drought years with a mean growing season average of 730 ± 123 mm. Thereby yields for the first cut were equal to non-drought years but overall lower yields were simulated for the second to the fifth cut while unfavorable growth conditions in drought years did not support a sixth cut as simulated for non-drought years.

## 4 Discussion

### 4.1 Capability of LandscapeDNDC to reproduce grassland cutting events and yields

Simulated grassland biomass production at different cutting dates over a 7-years observation period including the drought year 2018 agreed in sufficient accuracy with measurements and reproduced the seasonal pattern of the biomass dynamics as expressed by values of model performance measures ranging between 0.52−0.61 for r², 720−917 kg DW ha^−1^ for RMSE and 31.7–37.1 % for NRMSE. However, LandscapeDNDC failed to reproduce the exceptional high yields of first cuts in 2013. Since environmental conditions at this time were not different to other years it is difficult to evaluate to what degree this deviation is driven by uncertainty of measurements as represented by high standard deviations or by model parameterisation. Nevertheless, the comparable high agreement of simulated and measured yields is underlined by comparing our model performance measures with those of a multi-model ensemble approach for nine different grazed and mowed grassland sites across Europe from Sándor et al. (2017). In this study the majority of simulations revealed r² < 0.3 (maximum = 0.6) and NRMSE values for similar pre-alpine and alpine grassland sites in Switzerland, France and Germany ranging between 32.7 and 72.1 %. Another ensemble modelling approach for predominantly grazed permanent grassland resulted in NRMSE values for predicted yields in the majority of cases > 40 %, independent of the calibration intensity (Ehrhardt et al., 2018). Results of further single modelling studies underline the good performance of our LandscapeDNDC biomass simulations: Liebermann et al. (2020) simulated different CO_2_ scenarios for a mown grassland site in central Germany with LandscapeDNDC with overall RMSE values of cutting yields between 1010 and 1243 kg DW ha^−1^ from 1995 to 2011. An overall cutting yield RMSE of 1400 kg DW ha^−1^ with the PROGRASS model for a four-year long simulation period of a mowed grass-clover sward was documented by Lazzarotto et al. (2010). De Bruijn et al. (2012) simulated harvested biomass for an intensive managed grassland site in central Switzerland using the Oensingen Grassland Model (OGM) from 2002 to 2010, obtaining r² and NRMSE values of 0.54 and 22 %, respectively.

The ability of LandscapeDNDC to simulate biomass yields in good agreement with field measurements was essential for the implementation and application of the dynamic management approach, which automatically executes grassland cuts if the simulated biomass equals a defined target biomass. The target biomass was in our case best represented, successively determined during calibration, by a linear regression of the 75^th^ percentile of the observed yields against DOY, referenced as the site-specific approach. Since this approach requires substantial field data, we further tested a generic approach calculating biomass thresholds for cutting events by relative contributions of single cuts to estimated annual yields. With both approaches, the LandscapeDNDC model was able to simulate the timing of cutting events accurately, for both the calibration as well as the validation period, with r² > 0.89 in all cases. Due to limited availability of grassland management data, the dynamic management module was developed and validated on the same two grassland sites. For the general approach, one additional grassland site with measurements on cutting DOY and yields was taken into account. Despite this limitation, further tests of the general approach with long term (26 years) DWD observations of the day of first cut at three independent grassland sites in the pre-alpine region of South Germany revealed a robust transferability of the general approach to larger areas with comparable site and climate conditions. This would include larger pre-alpine regions in Austria and Switzerland. The robustness was represented by an average difference between simulated and observed cuts of 2.2 ± 7.5 days, even though detailed data on biomass, soil and vegetation were missing for those sites. Nevertheless, more thorough testing with additional field data will be necessary to further evaluate the transferability of the model to other sites and regions of concern.

The stronger systematic bias between the simulated and observed start of the growing season for the three DWD sites might be attributed to different definitions of this event. While criteria of observed dates are rather subjective and represented by 25 % of the grassland showing fresh green leaves irrespective of the species composition, the start of the growing season in the LandscapeDNDC model is strictly defined by a growing degree-day threshold.

In our approach a cutting event is executed latest after DOY 150 (1^st^ cut) or latest 55 days after the previous cutting event, whenever the target biomass is not reached. In implementing this rule, we considered not only physiological but also fodder quality aspects, because local farmers limit the time between single cuts to avoid loss of fodder quality due to too long ageing of the sward. In view of climate change and an earlier start of the growing season, the now strictly defined latest DOY for the first cut and the fixed days between cuts could be adapted by setting time limits for cutting events after certain days without further grassland growth (Vuichard et al., 2007) or for the first cut as a maximum number of days after the start of the growing season. However, keeping the maximum date for the first cut at DOY 150 did not yet cause any substantial bias in our simulations since this threshold was hardly reached, and if, only at Fendt at the beginning (2011−2025) of the simulation period (Fig. 5).

Despite the fact, that we derived our management rules from current data, this had no negative effect on the logic of simulated management operations and associated yield predictions under climate change conditions. Earlier cutting dates following from an earlier start of the growing season and better growth conditions because of higher temperature and atmospheric CO_2_ correspond to a higher target biomass as calculated from the regression with DOY. Furthermore, earlier cutting dates in combination with the expanding of the growing season in autumn allow for an increasing number of total cutting events per year from currently four to five or even six, which also support higher yields under climate change conditions (Höglind et al., 2013; Thivierge et al., 2016). Accordingly, predicted yields for a temperature increase of +2 °C are about 6–12 % higher than current yields in both scenarios RCP 4.5 and 8.5. This agrees well with field observations of a climate warming experiment with intact grassland monoliths from the Graswang site (Fu et al., 2019).

So far, the timing of grassland cutting events in climate change modelling studies mainly relied on rule sets using cumulative growing degree days (GDD) (Höglind et al., 2013; Jing et al., 2014, 2013; Thivierge et al., 2016). However, these only temperature informed GDD based approaches do not allow considering stimulation of plant growth by increasing atmospheric CO_2_ concentration and limitation of plant growth under drought conditions, particularly in spring and summer (Chang et al., 2017; Ganjurjav et al., 2016). A more detailed GDD approach was developed within the STICS model (Brisson et al., 2003), where cutting events can be defined by the user in the form of GDD. Once the GDD have been reached, cutting is triggered only if the harvestable biomass (above a certain height of grass) exceeds a minimum value set by the user. Otherwise cutting is shifted until the model can at least harvest this minimum biomass. Cutting is therefore conditioned by grass growth which itself depends in particular on the atmospheric CO_2_ concentration and the availability of soil water and nutrients. This approach has been used by Juin et al. (2004) for climate change scenario simulations for a permanent alpine grassland in France, supporting our findings of earlier first cutting dates and increasing number of cutting events by 2070−2100. As drought is expected to become more frequent and more intense under climate change in the pre-alpine region (Gobiet et al., 2014; Samaniego et al., 2018), physiological based rules sets as those presented here can have advantages over solely temperature driven growth relationships. This was underlined from our simulations by 15 % lower simulated yields in drought years (Fig. 8). Though intense spring and summer drought reduced yields from the second cut onwards, yields at first cutting events were not influenced due to the high soil water holding capacities in combination with significant amounts of water from precipitation and snow melt filling up the soil profile. Due to the high contribution of the first cut, this makes annual grassland yields in our study region likely less sensitive to climate change than in regions with lower precipitation and more unfavorable soil conditions.

Our approach is fully dynamic and calculates cutting events on the fly of biogeochemical simulations without the need of file-based changes of harvesting schemes as applied by Thivierge et al. (2016) or the calculation of the number of yearly cuts in advance of simulations (Höglind et al., 2013). To estimate the target biomass our general approach requires a minimum of mandatory user input, e.g. site altitude, but can be specified also by more detailed field observations in the site-specific mode. An alternative approach to automatically derive management events is the optimal management algorithm proposed by Vuichard et al. (2007) which maximizes the seasonal dry matter production. With this approach, scheduled cutting events are coupled to plant growth by triggering a cut after a minimum of 30 days of regrowth and declining plant growth rates during 10 consecutive days. This was widely used to automatically derive management operations for site and regional simulations (Chang et al., 2015; Gómara et al., 2020; Vuichard et al., 2007), and within regional climate change assessment studies (Chang et al., 2017). So far these studies applied a rather coarse validation scheme by comparing simulated yields with data from Europe wide yield databases (Chang et al., 2015; Vuichard et al., 2007; Rolinski et al., 2018). To our knowledge our study is the first that validates an automatically adaptive management routine against detailed field data and phenological observations (e.g. first cutting DOY). Our results show that a detailed model validation is of high relevance since even small changes in cutting frequency (one cut/year difference) can significantly influence annual yield predictions particularly under climate change conditions.

### 4.2 Impact of climate change on grassland cutting dates and yields

Climate change shifts the beginning of the vegetation period, allowing for earlier first cutting dates. Mean absolute changes over all ten different climate realizations between the period 2011−2040 and 2071−2100 were 18 ± 1.17 days and 37 ± 1.6 days for the RCP 4.5 (+1.7 °C) and RCP 8.5 (+4.4 °C) scenario, respectively, with minor differences between the Fendt and Graswang sites. This resulted in increasing number of cuts, from four to five under present conditions, to constantly five cutting events per year after 2035, and even six in the RCP 8.5 scenario after 2080 at both sites. Based on the DWD phenological data we were able to show that the observed rates of shifted first cutting events related to the observed air temperature increase agreed very well with simulations (Table 5). While the reduction of yearly nitrogen fertilization to a maximum of 170 kg ha^−1^ yr^−1^ had no impact on the DOY of the first cut, overall lower number of cutting events associated with reduced nitrogen fertilization rates slowed down regrowth which resulted in longer periods between single cutting events and lower yields as compared to the non-reduced fertilization scenario.

Overall, the simulated shifts of the first cutting event compares well with other observations. Deroche et al. (2020) reported a 14-day earlier start of the vegetation period for a grassland site (900−1200 m.a.s.l.) in France for a +1 °C air temperature increase in the period 1979–2010. Results from Parmesan and Yohe (2003) for recent species’ advancement of spring events of 2.3 days per decade and increase of the length of the growing season of +0.261 ± 0.008 days per year presented by Menzel et al. (2020) would translate into 20.7 and 23 days until 2100 respectively, which fits well with our simulated first cutting shifts under the RCP 4.5 scenario.

The increasing uncertainty of our results over time must be taken into account when discussing the implications, especially the strong shift of the first cut at the end of the simulation period under the RCP 8.5 scenario. In addition to the uncertainty associated to climate projections (IPCC, 2014; Knutti and Sedláček, 2013), there are uncertainties associated with exceeding boundary conditions of current process descriptions of biogeochemical models. For instance, climate change could modify overwintering mechanisms (Ergon et al., 2018; Katata et al., 2020), leading to altered plant storage dynamics and thus altered spring growth (Rapacz et al., 2014). Considering these uncertainties, the first cutting dates in Fendt at the end of the century (DOY 72 = 13^th^ of March) in the high-emissions scenario RCP 8.5 appear debatable, particularly because they entail radiation intensities that are low for supporting plant growth (Höglind et al., 2013). However, these mechanisms are very complex and not fully understood (Höglind et al., 2011; Wingler and Hennessy, 2016), especially those regarding resource-acquisition-, assimilation- and overwintering abilities of different grassland species, and those related to community dynamics, with more thermophilic grassland communities likely to be found more often under climate change conditions (Fridley et al., 2016). Since 2011 changes in plant species composition and therefore changes in functional diversity at the Graswang and Fendt site were minor (unpublished data), thus should not have a high impact for the RCP 4.5 scenario simulations. This is likely different for the RCP 8.5 scenario with more severe changes of environmental parameters so that uncertainty associated with species composition changes of simulations should be higher, too. However, sound adaptation of grassland growth parameters is still a major problem and highly hampered since data available from grassland warming experiments and increasing atmospheric CO_2_ is still low and findings often contradicting (Ghahramani et al., 2019; Wang et al., 2019).

Modelling studies of climate change impacts on grasslands in Canada based on GDD-based management show weaker shifts of the first cutting dates of −5 to −3.2 days per +1 °C temperature increase (Jing et al., 2014, 2013; Thivierge et al., 2016). Absolute shifts in Northern Europe of 22 days between the reference and the climate change period are reported by Höglind et al. (2013). Two aspects may explain the lower temperature sensitivity of these high latitude sites. First, an overall lower MAT + 4 °C (Jing et al., 2013), which can limit growth under future climatic conditions in spite of more pronounced temperature increase (+1−2 °C higher than in this study). Second, differences in grassland species dominance, with a predominance of timothy (Phleum pratense L.) which is better adapted to colder temperatures (Jing et al., 2013) but less productive with respect to regrowth capacity as ryegrass dominated temperate grasslands (Höglind et al., 2010, 2013). Nevertheless, the increase in cutting intensity of up to two additional cuts is coherent across different study regions (Höglind et al., 2013; Jing et al., 2014; Thivierge et al., 2016).

Compared to the static management, up to 20 % higher yields (2000 kg DW ha^−1^ yr^−1^) were simulated with the dynamic management without nitrogen reduction particularly in the RCP 8.5 scenario at the end of the simulation period. This resulted from both, higher yields at respective cutting events and increased number of cuts per year. In contrast to Höglind et al. (2013), contributions of an additional 6^th^ cut (∼ 250 kg DM ha^−1^ yr^−1^) were less important at our study sites (Fig. 8). Considering all scenarios, yield increases induced by climate change were higher in the RCP 8.5 scenario (up to 2000 kg DW ha^−1^ yr^−1^) than in the RCP 4.5 scenario (up to 650 kg DW ha^−1^ yr^−1^). These values compare well with increases of yields reported in other grassland simulation studies based on GDD based management approaches, with yield differences between static and dynamic management in the same order of magnitude (Jing et al., 2014; Thivierge et al., 2016).

Climate change increases in yields can be related to increasing air temperature and atmospheric CO_2_ concentration. As outlined in Fig. 8, yield stimulation can be offset by drought stress, which in our case is more pronounced at the Fendt site (Fig. 7) because of higher MAT, lower MAP and lower water retention in the sandier soils (Table 1, Table 2). For the same reasons yield increases with climate change (both RCP 4.5 and RCP 8.5) were generally lower than at Graswang (Fig. 7). Variability of yields did not significantly increase with climate change since growing season precipitation (Table 3) even for the period 2071−2100 at the drier Fendt site mostly exceeded the amount of 550 mm found as limit for reduction in yields (Fig. 8). Although Ruelle et al. (2018) predicted higher yield variabilities between years and stronger reductions in yields with severe climate change for pastures in Ireland with adapted grazing events and comparable soils and weather, overall pasture yields and forage production in the Alpine and northern region is, in line with our results, predicted to increase due to longer growing seasons and still sufficient water availability (Dellar et al., 2018; Höglind et al., 2013).

Interestingly, yield increases at both sites and particularly for the RCP 8.5 scenario are most pronounced for simulations with the dynamic non-reduced N management but less (Graswang) or even non evident (Fendt) with static management. This is in line with Thivierge et al. (2016) who also found that climate adapted management can compensate for unfavorable growth conditions and can lead to an increase of annual yields which could not be achieved with static management settings derived from current climate conditions.

Within our dynamic management approach without nitrogen reduction, increasing number of cuts also led to increasing number of manure events, thus higher loads of N fertilization (up to 280 kg N ha^−1^ yr^−1^), particularly towards the end of the RCP 8.5 simulation period, while annual fertilization rates in the static management remain lower at 190 kg N ha^−1^ yr^−1^. Yields of the dynamic reduced nitrogen fertilization scenario (maximum of 170 kg ha^−1^ yr^−1^) were in a comparable range than those of static management and thus significantly lower than simulated with the dynamic non-reduced nitrogen management. This shows that potential yield increases under climate change conditions can only be achieved if also manure application rates are adjusted. This is in line with Lee et al. (2013), showing that under lower nitrogen availability the growth-promoting effects of climate change could not be fully exploited. However, higher fertilization rates are conflicting with current regulations of the German Fertilizer Ordinance (DüV), which limits average annual N fertilization rates of the farm's utilized agricultural areas to 170 kg N ha^−1^ yr^−1^ (Achilles et al., 2018). As reported from measurements, environmental nitrogen losses of the studied grasslands e.g. in form of N_2_O (Zistl-Schlingmann et al., 2019) and nitrate leaching (Fu et al., 2017) even with fertilization rates of up to 300 kg N ha^−1^ yr^−1^ were rather low. Thus, careful intensification of grassland management to support increased yields under climate change at suitable fields could allow for reducing management intensity at other fields supporting e.g. biodiversity and likely allowing improved ecosystem service provision on farm and regional scale.

## 5 Conclusions

Our study demonstrates the importance of dynamic rules for adapting management activities to changing environmental conditions in the context of model-based assessments of climate change impacts on grassland productivity. This finding highly supports the statement of Kipling et al. (2016) that the application and validation of different management strategies suitable for climate change conditions remains a key challenge for modelling studies targeting European grassland systems. We successfully implemented our dynamic management module with a focus on pre-alpine grassland systems, yet we argue that setting cuts on the fly of simulations is feasible also for other regions of concern. The findings that positive effects of climate change on grassland productivity are contingent on increasing number of cuts and rates of N fertilization and the possibility to mitigate the negative impacts of drought, clearly call for a weather driven optimization of grassland management operations. Taking into account that climate variability is expected to further increase, we conclude that grassland management decision making is likely to get more and more challenging. As climate adapted management leads to more frequent cutting and manuring events and thus higher trafficability, constraints linked to soil bearing capacity and labour need to be taken into account since they may limit implementation. By providing means to test different adaptation measures, simulation models such as LandscapeDNDC can be crucial in informing sustainable use of grassland systems and related socio-economic consequences in the long term.

## CRediT authorship contribution statement

**Krischan Petersen**, **David Kraus**, **Pierluigi Calanca** contributed to further development of the model; **Krischan Petersen** and **Mikhail A. Semenov** developed the climate change scenario data; **Krischan Petersen**, **Pierluigi Calanca** and **Ralf Kiese** planned and carried out the simulations; **Krischan Petersen** and **Ralf Kiese** took the lead in writing the manuscript with input from all authors. All authors provided critical feedback and helped shape the research, analysis and manuscript. **Krischan Petersen** and **Klaus Butterbach-Bahl** conceived the study and were in charge of overall direction and planning.

## Declaration of Competing Interest

The authors report no declarations of interest.
