# Supplementary material for: Dynamic simulation of management events for assessing impacts of climate change on pre-alpine grassland productivity
Source: Eur J Agron. 2021 Aug;128:None. doi: 10.1016/j.eja.2021.126306 (PMC8209143; doi:10.1016/j.eja.2021.126306)
Supplement: Supplementary file 1 [file mmc1.docx]

**Supplementary material for**

**Dynamic simulation of management events for assessing impacts of climate change on pre-alpine grassland productivity**

Petersen, K. et al.


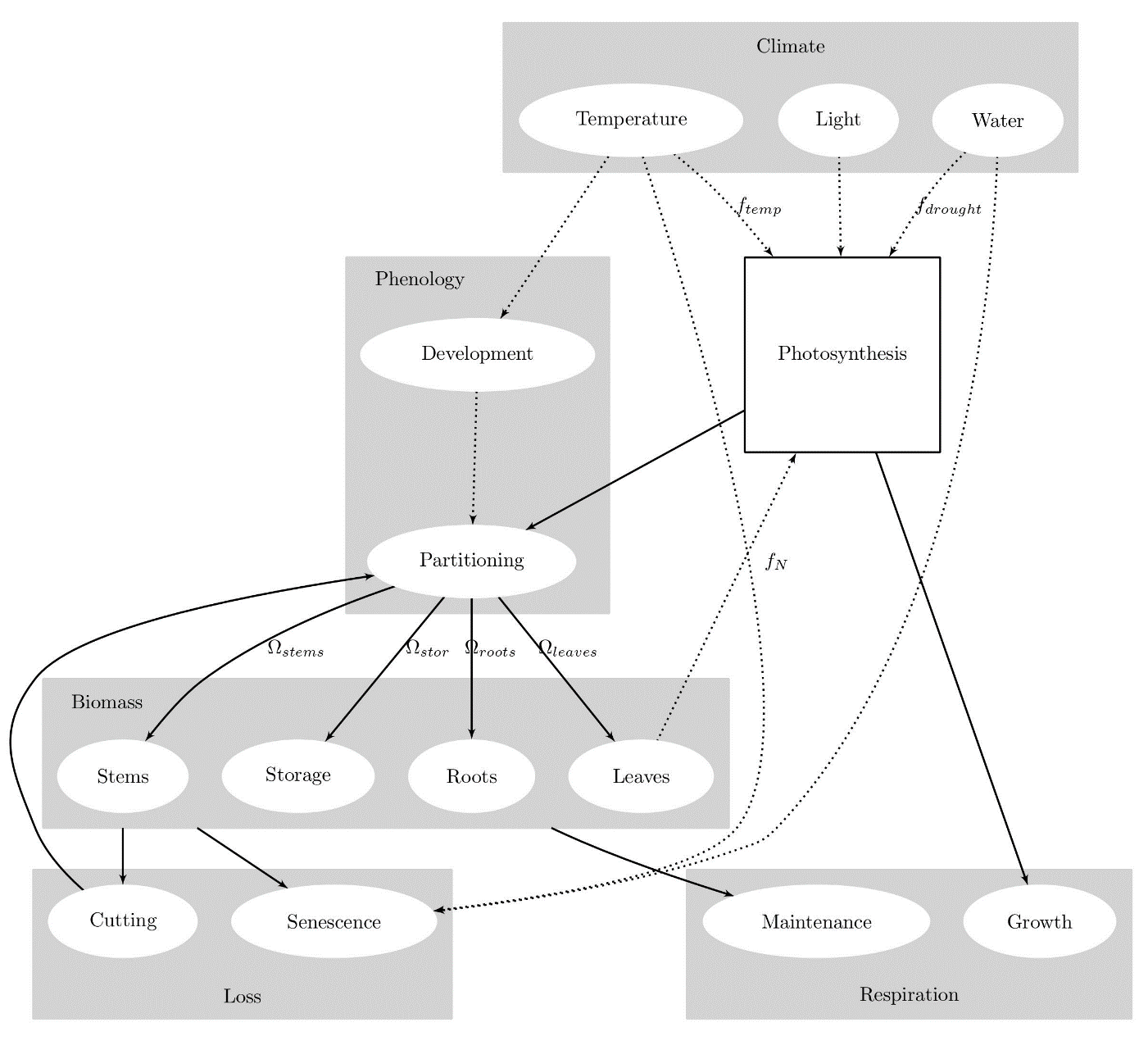


Figure S1: Flow chart of the plant physiology model Plamo^x^. $\Omega_{x}$ defines species-specific parameters; $f_{x}$ defines response functions for the influence of drought, temperature and nitrogen on photosynthesis.

|  |  | **BD** | **pH** | **C_org_** | **N_org_** | **Clay** | **Silt** | **Sand** | **Stone** | **FC (pF 1.8)** | **PWP (pF 4.2)** | **HC** |
| --- | --- | --- | --- | --- | --- | --- | --- | --- | --- | --- | --- | --- |
| **Sites** | **Depth** | g kg^-1^ |  | Weight-% | | Fractions (%) | | | | Vol.-% | | cm min^-1^ |
| **Graswang** | 0 - 5 | 0.6 | 4.9 | 10.0 | 1.0 | 58.5 | 35.1 | 6.4 | 1.0 | 52.0 | 22.1 | 0.005 |
|  | 5 - 16 | 0.8 | 7.1 | 5.8 | 0.7 | 58.5 | 35.1 | 6.4 | 1.5 | 52.0 | 22.1 | 0.005 |
|  | 16 - 37 | 1.2 | 7.6 | 2.0 | 0.3 | 59.2 | 35.9 | 4.9 | 0.0 | 50.0 | 17.5 | 0.005 |
|  | 37 - 46 | 1.4 | 7.5 | 1.0 | 0.1 | 55.2 | 41.3 | 3.5 | 0.0 | 43.0 | 13.5 | 0.005 |
|  | 46 - 53 | 1.2 | 7.4 | 1.6 | 0.2 | 60.5 | 38.5 | 1.0 | 0.0 | 45.0 | 21.7 | 0.004 |
|  | 53 - 72 | 1.4 | 7.5 | 0.7 | 0.1 | 57.2 | 39.7 | 3.1 | 0.0 | 42.1 | 11.2 | 0.005 |
|  | 72 - 86 | 1.4 | 7.6 | 0.5 | 0.1 | 55.6 | 37.4 | 7.0 | 0.0 | 40.0 | 10.1 | 0.006 |
|  | 86 - 109 | 1.5 | 7.6 | 0.4 | 0.1 | 53.0 | 43.3 | 3.7 | 0.0 | 41.0 | 7.8 | 0.005 |
|  | 109 - 124 | 1.5 | 7.6 | 0.4 | 0.0 | 54.0 | 42.0 | 4.0 | 0.0 | 42.6 | 9.3 | 0.005 |
|  | 124 - 143 | 1.5 | 7.7 | 0.4 | 0.0 | 54.3 | 43.2 | 2.5 | 0.0 | 42.6 | 16.1 | 0.005 |
| **Fendt** | 0 - 5 | 0.7 | 5.1 | 6.8 | 0.7 | 27.2 | 40.3 | 32.5 | 0.0 | 50.0 | 23.5 | 0.020 |
|  | 5 - 12 | 1.1 | 6.6 | 4.4 | 0.5 | 25.2 | 40.3 | 34.5 | 3.8 | 46.0 | 23.5 | 0.020 |
|  | 12 - 27 | 1.1 | 6.4 | 1.8 | 0.2 | 25.2 | 40.0 | 34.8 | 5.8 | 45.0 | 20.1 | 0.023 |
|  | 27 - 44 | 1.4 | 6.6 | 0.8 | 0.1 | 36.5 | 33.9 | 29.6 | 10.7 | 45.0 | 28.1 | 0.016 |
|  | 44 - 80 | 1.6 | 7.4 | 0.5 | 0.0 | 30.1 | 44.6 | 25.3 | 41.8 | 23.1 | 15.1 | 0.022 |
|  | 80 - 108 | 1.6 | 7.8 | 0.2 | 0.0 | 30.6 | 29.9 | 39.5 | 48.2 | 20.1 | 13.3 | 0.024 |
|  | 108 - 123 | 1.6 | 7.7 | 0.2 | 0.0 | 35.3 | 41.8 | 22.9 | 9.7 | 30.8 | 15.2 | 0.012 |

Table S1: Physical and chemical soil characteristics of the grassland sites Fendt and Graswang as used for the simulations; BD = bulk density, C_org_ = organic carbon content, N_org_ = organic nitrogen content, FC = field capacity, PWP = permanent wilting point, HC= hydraulic conductivity.

| **MS parameter** | **EQ Nr.** | **LDNDC parameter** | **Value setting** | **Description / Unit** |
| --- | --- | --- | --- | --- |
| Ω_SLA_ |  | SLAMAX* | 16 | Specific leaf area (m² kg^-1^) |
| Ω_STORAGE_ | 1 | FRACTION_FRUIT | 0.15 | Parameter controlling allocation to fruit/storage compartment |
| Ω_ROOT_ | 4 | FRACTION_ROOT | 0.4 | Parameter controlling allocation to root compartment |
| Ω_LEAF_ ** | 4, 6 | FRACTION_FOLIAGE | 0.36 | Parameter controlling allocation to foliage/leaf compartment |
| γ_CUT_ | 4, 5 | Y_CUT | 0.25 | Factor to increase the allocation to aboveground biomass before the first cut |
| Ω_GDD_ | 2 | GDD_MATURITY | 2500 | Growing degree days for full plant development |
| Ω_T,BASE_ | 3 | GDD_BASE_TEMPERATURE | 0 | Base temperature for the increment of GDD |
| Ω_NC,STORAGE_ | 7 | NC_FRUIT_MAX | 0.03 | Optimum nitrogen concentration of fruit/storage (kg kg^-1^) |
| Ω_NC,ROOT_ | 7 | NC_FINEROOTS_MAX | 0.028 | Optimum nitrogen concentration of fine roots (kg kg^-1^) |
| Ω_NC,LEAF_ | 7, 11 | NC_FOLIAGE_MAX | 0.028 | Optimum nitrogen concentration of foliage (kg kg^-1^) |
| Ω_NC,STEM_ | 7 | NC_STRUCTURAL_TISSUE_MAX | 0.028 | Optimum nitrogen concentration of straw/stems (kg kg^-1^) |
| Ω_LIMIT_ | 10 | TLIMIT | 5 | Temperature limit for plant growth (°C) |
| Ω_RUBISCO_ | 8 | VCMAX25 | 70 | Maximum RubP saturated rate (Rubisco activity) of  carboxylation at 25oC for sun leaves (umol m² s^-1^). |
| Ω_H2O;_ Ω_H2O,SEN_ | 9, 16 | H2OREF_A | 0.5*** | Relative available soil water content at which stomata conductance is affected |
| Ω_NDEF,LEAF_ | 11 | N_DEF_FACTOR | 2 | Factor defines nitrogen deficiency |
| Ω_YIELD_ | 12 | FYIELD | 0.25 | Fraction of growth respiration relative to gross assimilation |
| Ω_LEAF_ | 13 | MC_LEAF | 0.03 | Maintenance respiration coefficient of leaves |
| Ω_ROOT_ | 13 | MC_ROOT | 0.01 | Maintenance respiration coefficient of roots |
| Ω_STEM_ | 13 | MC_STEM | 0.015 | Maintenance respiration coefficient of stems |
| Ω_STORAGE_ | 13 | MC_STORAGE | 0.001 | Maintenance respiration coefficient of storage organs |
| Ω_T,REF_ | 13 | MAINTENANCE_TEMP_REF | 25 | Reference temperature for maintenance respiration (°C) |
| Ω_EXUDATE_ | 14 | DOC_RESP_RATIO | 0.5 | Ratio of root exudates related to root growth respiration |
| Ω_SEN,DROUGHT_ | 16 | SENESCENCE_DROUGHT | 0.01 | Coefficient of senescence related to aging |
| Ω_SEN,FROST_ | 17 | SENESCENCE_FROST | 0.01 | Coefficient of senescence related to frost |
| Ω_SEN,AGE_ | 18 | SENESCENCE_AGE | 0.003 | Coefficient of senescence related to age |
|  |  |  |  |  |
| *: LandscapeDNDC generally includes two parameters for the description of specific leaf area, i.e., SLAMAX and SLAMIN representing leaf area under full light (top of canopy) and shaded conditions (lower canopy depth), respectively. This distinction, however, is not considered by PlaMox, which assumes a vertically homogeneous mean specific leaf area that is provided for practical reasons through the parameter SLAMAX. Hence, as in this application SLAMAX does not indicate an upper limit, its value is lower than maximum parameter values found otherwise in the literature. | | | | |
| ****** : Ω_STEM_ (FRACTION_STEM) corresponds to 1 - Ω_FRUIT_ - Ω_ROOT_ - Ω_FOLIAGE_ | | | |  |
| ***: This parameter is set for the Fendt site to 0.4 to account for more drought tolerant species | | | | |

Table S2: LandscapeDNDC vegetation growth parameters for parametrized grassland simulations. From left: Parameter name used in the manuscript, equation number in the manuscript in which parameter is used, internal LandscapeDNDC (Plamox) parameter name, parametrized simulation value for all sites, process-related description.


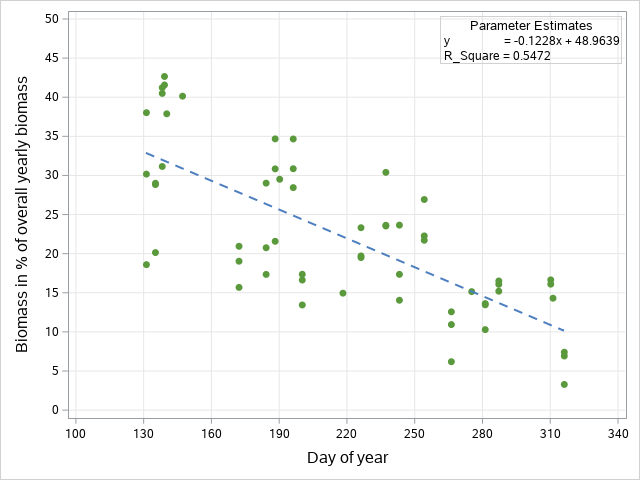


Figure S2: Fitted regression function for relative contribution (in %) of each cutting event to the annual biomass production from three grassland sites (general regression approach); each point bases on mean of three replicates at each cutting event.
